# Neuroplasticity and Healthy Lifestyle: How Can We Understand This Relationship?

**DOI:** 10.1155/2017/9506181

**Published:** 2017-10-16

**Authors:** Azucena Begega, Luis J. Santín, Pablo Galeano, Debora Cutuli, Patricia Sampedro-Piquero

**Affiliations:** ^1^Neuroscience Institute (INEUROPA), Universidad de Oviedo, Oviedo, Spain; ^2^Universidad de Málaga, Málaga, Spain; ^3^Consejo Nacional de Investigaciones Científicas y Técnicas (CONICET), C1425FQB Buenos Aires, Argentina; ^4^University of Rome, Rome, Italy; ^5^Universidad Autónoma de Madrid, Madrid, Spain

Our brain has this extraordinary ability to experience functional and structural changes before environmental stimuli, cognitive demand, or our experience itself. Exercise, diet, an appropriate sleep pattern, and reading habits are among those activities proposed to induce effects on cerebral architecture—an active lifestyle seems to induce changes in the brain function that favour welfare and better quality of life. This special issue is intended to extend the knowledge about the relationship between neuroplasticity and a healthy lifestyle.

L. Mandolesi et al. present a broad approach on environmental effects on neural plasticity in “Environmental Factors Promoting Neural Plasticity.” Combining concepts such as brain reserve and cognitive reserve allows us to understand how different lifestyles impact both brain architecture and function. Therefore, physical activity, an appropriate sleep pattern, and certain diet are thought to promote better cognitive functioning, leading to a reduction of deficits such as those associated with ageing. In this sense, C. Phillips shows us how to achieve both physical and psychological health in her review “Lifestyle Modulators of Neuroplasticity: How Physical Activity, Mental Engagement, and Diet Promote Cognitive Health during Aging.”

Physical activity has been proposed as a modulator of brain activity and cognition throughout the lifespan. Y.-K. Chang et al., in their study “Exercise Modality Is Differentially Associated with Neurocognition in Older Adults,” conclude that both aerobic exercise and a programme of coordinated exercise have beneficial effects in the Stroop test for inhibitory control in individuals ranging from 55 to 70 years old—both groups exhibit lower reaction time in the Stroop test. After ERP (event-related potential) analysis, the authors highlight how N450 wave is reduced in exercised subjects, which could be reflected in the reduced activity in the anterior cingulate cortex, a brain area related to conflict resolution processes. The lower amplitude in N450 wave might indicate higher resolution capacity in the Stroop test. In line with this work, C.-H. Chu et al. in “Acute Exercise and Neurocognitive Development in Preadolescents and Young Adults: An ERP Study” propose that a simple exercise programme (20 mins) in preadolescent and young adults improves the performance in the Stroop test. The ERP technique showed an increase in P300 wave in every exercised group, accompanied by an improvement in control inhibition processes measured in the Stroop test.

On the other hand, M. Tajerian and J. D. Clark thoroughly review alternative medicine interventions in “Nonpharmacological Interventions in Targeting Pain-Related Brain Plasticity.” Their analysis include a review of not only several of these interventions (distraction, mindfulness and meditation, cognitive behaviour therapies, etc.) but also the plasticity mechanisms underlying each one of them. Although there are no conclusive data, it seems that alternative therapies could be a great complementary tool to classic pharmacological interventions.

Stressors are among the neuroplasticity-affecting agents that could disfavour welfare, as stressful events can induce negative effects on both cerebral and cognitive functioning. M. S. Henry et al. propose that higher resilience could reduce negative stress-related outcomes in their review “Enkephalins: Endogenous Analgesics with an Emerging Role in Stress Resilience.” The concept of resilience is referred to as the ability an individual has to adapt to adverse conditions that could happen in life. It is a complex process combining coping abilities with neurobiological processes and the interaction between them. The study of resilience is likely to extend our understanding of affective disorders such as depression and anxiety.

In the past few years, it has been proposed that healthy habits (exercise, diet, etc.) could promote resilience. M. S. Henry et al. conclude that enkephalins (ENK) could play a relevant role in promoting resilience, increasing the subject's adaptability to the environment. Taking into account the distribution of enkephalins and their receptors in the hippocampus, the amygdala (AMG), the medial prefrontal cortex (mPFC), and the nucleus accumbens (NAc), these opioid neurotransmitters are proposed not only to exert analgesic effects but also to affect emotional responses; the level of anhedonia in rats, measured in the sucrose preference test, is increased when ENK transcripts show a reduction in the basolateral amygdala. M. S. Henry et al. show that resilience to social defeat and chronic unpredictable stress share common variations of expression among enkephalin systems within specific brain regions in rats. ENK mRNA (transcripts) were quantified in 23 nuclei of the mPFC, NAc, dorsal striatum, and AMG. Only one significant difference between control, resilient, and vulnerable individuals was found in the BLA of vulnerable individuals; ENK mRNA levels were decreased in vulnerable rats compared to control and resilient rats. This work extends the action of enkephalins to regions like the preoptic area and the bed nucleus of the stria terminalis (BST) with regard to stress resilience. Hence, modulating ENK or DOPr/MOPr expression within circumscribed regions or modulating selected neuronal circuits appears to be more appropriate.

Finally, X. Chen et al. analysed in their research “The Rapid Effect of Bisphenol-A on Long-Term Potentiation in Hippocampus Involves Estrogen Receptors and ERK Activation” the effects of bisphenol on memory-related processes such as long-term potentiation (LTP). Bisphenol-A (BPA) is a widely used synthetic compound included in polycarbonate plastics and epoxy resins, for example, in food and beverage containers, dental prostheses, compact discs, and baby bottles. Its action on the endocrine function has been assessed in numerous studies, showing that low doses can inhibit sexual differentiation and lead to relevant outcomes during adulthood. X. Chen et al. show that bisphenol-A exerts dose-dependent effects; that is, they observe biphasic effects of low-dose (100 nM) and high-dose (1000 nM) BPA on hippocampal LTP. The exposure to BPA at a low dose enhanced LTP, while exposure to a high dose inhibited LTP compared with vehicle controls. These effects require the participation of the membrane-associated estrogen receptor (ER).

We have tried to include in this special issue those studies analysing the role of healthy habits and how brain plasticity can be affected by them. The aim of this special issue was to give an insight into the current research of promoting quality of life.

